# Bis(hy­droxy­ammonium) hexa­chlorido­platinate(IV)–18-crown-6 (1/2)

**DOI:** 10.1107/S1600536813032649

**Published:** 2013-12-07

**Authors:** Evgeny Bulatov, Anastasiya Afanasenko, Tatiana Chulkova, Matti Haukka

**Affiliations:** a Department of Chemistry, Saint Petersburg State University, Universitetsky Pr. 26, 198504 Stary Petergof, Russian Federation; b Department of Chemistry, University of Jyvaskyla, PO Box 35 FI-40014 Jyvaskyla, Finland

## Abstract

In the title complex, (NH_3_OH)_2_[PtCl_6_]·2C_12_H_24_O_6_, the Pt^IV^ atom is coordinated by six chloride anions in a slightly distorted octa­hedral geometry. The Pt—Cl bond lengths are comparable to those reported for other hexa­chlorido­platinate(IV) species. The hy­droxy­ammonium groups act as linkers between the [PtCl_6_]^2−^ anion and the crown ether mol­ecules. The anion is linked to two hy­droxy­ammonium cations *via* O—H⋯Cl hydrogen bonds and each hy­droxy­ammonium moiety is linked to a crown ether mol­ecule by hydrogen bonds between ammonium H atoms and 18-crown-6 O atoms. The crown ether mol­ecules have the classic crown shape in which all O atoms are located in the inner part of the crown ether ring and all –CH_2_– groups are turned to the outside.

## Related literature   

For general background to supra­molecular assemblies, see: Saalfrank & Demleitner (1999 ▶). For crystal structures of related compounds based on platinum complexes and crown ether mol­ecules, see: Bulatov *et al.* (2012 ▶).
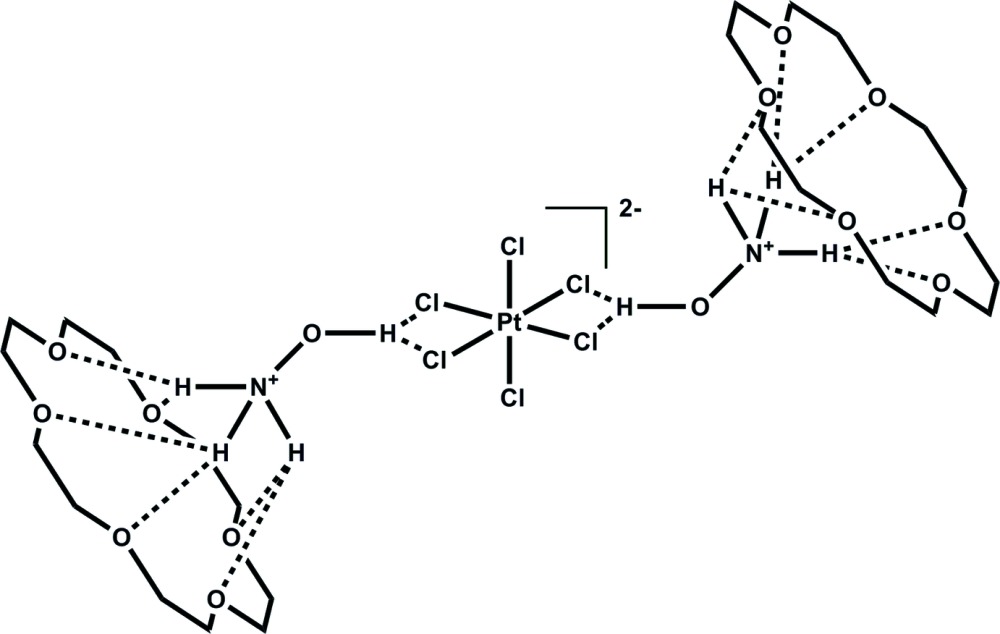


## Experimental   

### 

#### Crystal data  

(NH_4_O)_2_[PtCl_6_](C_12_H_24_O_6_)_2_
*M* *_r_* = 1004.50
Orthorhombic, 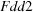

*a* = 29.6079 (10) Å
*b* = 30.5302 (10) Å
*c* = 8.5175 (3) Å
*V* = 7699.3 (5) Å^3^
*Z* = 8
Mo *K*α radiation
μ = 4.12 mm^−1^
*T* = 100 K
0.48 × 0.12 × 0.11 mm

#### Data collection  

Bruker Kappa APEXII DUO CCD diffractometer
Absorption correction: multi-scan (*SADABS*; Sheldrick, 2008*a* ▶) *T*_min_ = 0.241, *T*_max_ = 0.664
29930 measured reflections
4628 independent reflections
4037 reflections with *I* > 2σ(*I*)
*R*_int_ = 0.067

#### Refinement  

*R*[*F* ^2^ > 2σ(*F* ^2^)] = 0.033
*wR*(*F* ^2^) = 0.054
*S* = 1.13
4628 reflections
215 parameters
1 restraint
H-atom parameters constrained
Δρ_max_ = 1.49 e Å^−3^
Δρ_min_ = −1.23 e Å^−3^
Absolute structure: Flack (1983 ▶), 2066 Friedel pairs
Absolute structure parameter: 0.035 (6)

### 

Data collection: *APEX2* (Bruker, 2009 ▶); cell refinement: *SAINT* (Bruker, 2009 ▶); data reduction: *SAINT*; program(s) used to solve structure: *SIR97* (Altomare *et al.*, 1999 ▶); program(s) used to refine structure: *SHELXL97* (Sheldrick, 2008*b* ▶); molecular graphics: *DIAMOND* (Brandenburg, 1999 ▶); software used to prepare material for publication: *SHELXL97*.

## Supplementary Material

Crystal structure: contains datablock(s) I. DOI: 10.1107/S1600536813032649/pk2505sup1.cif

Structure factors: contains datablock(s) I. DOI: 10.1107/S1600536813032649/pk2505Isup2.hkl

Supporting information file. DOI: 10.1107/S1600536813032649/pk2505Isup3.cdx

Additional supporting information:  crystallographic information; 3D view; checkCIF report

## Figures and Tables

**Table 1:** Hydrogen-bond geometry (Å, °)

*D*—H⋯*A*	*D*—H	H⋯*A*	*D*⋯*A*	*D*—H⋯*A*
O7—H7*O*⋯Cl4	0.84	2.44	3.237 (4)	159
O7—H7*O*⋯Cl3	0.84	2.69	3.184 (4)	119
N1—H1*C*⋯O1	0.91	1.90	2.811 (5)	177
N1—H1*D*⋯O3	0.91	2.02	2.849 (5)	152
N1—H1*D*⋯O4	0.91	2.54	3.006 (5)	113
N1—H1*E*⋯O5	0.91	1.95	2.856 (6)	172
N1—H1*E*⋯O6	0.91	2.58	3.039 (5)	112

**Fig. 1.**
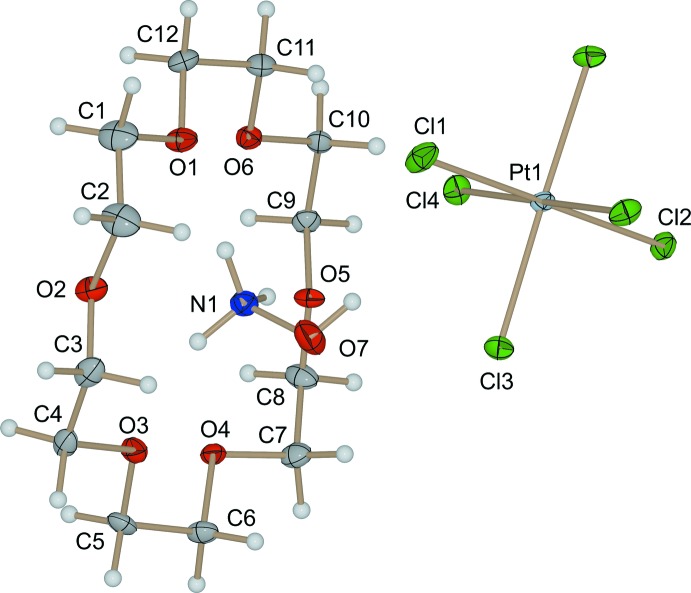
The molecular structure of the title compound with displacement ellipsoids drawn at the 50% probability level.

## supplementary crystallographic information

### 1. Comment

The crystal structure of the title complex contains one Pt atom coordinated by six Cl atoms in an octahedral geometry (Fig. 1). The Pt—Cl1, Pt—Cl3, and Pt—Cl4 distances are 2.328 (3), 2.3202 (10), and 2.3184 (10) Å, respectively. The hydroxyammonium ions act as linkers between the [PtCl_6_]^2-^ moieties and the crown ether molecules. The O—H···Cl and N—H···O hydrogen bond parameters are given in Table 1. Association with the platinum complexes changes the conformation of the crown ether. Thus, the cavity of the free 18-crown-6 has two inward-turned CH_2_ groups and two oxygens with the electron pairs facing outward and away from the center. In other words, the free crown ether does not display the true crown shape or cavity. However, in the presence of (NH_3_OH)_2_[PtCl_6_], reorganization of the crown occurs to give the classic crown shape in which all oxygen atoms are located in the inner part of the crown ring and all CH_2_ groups are turned to the outside.

### 2. Experimental

A mixture of cis-[PtCl_2_(HON=C(CH_3_)_2_)_2_] (0.045 mmol, 0.019 g) and N,N-dichlorotosylamide (0.090 mmol, 0.022 g) in chloroform (5 mL) was refluxed for 2 h, whereupon the reaction mixture was passed through a silica gel (60 Å; Merck) column using chloroform as the eluent. The resulting yellow solid was co-crystallized with 18-crown-6 in a 1:2 molar ratio from an acetone:chloroform (2:3, v/v) solution at 20–25 °C to give yellow crystals (yield 46%).

### 3. Refinement

The OH hydrogen atom was located in a difference Fourier map but refined with fixed distances and angles (O—H = 0.84 Å and N—O—H = 109.47°) using a riding model with U_iso_ = 1.5U_eq_ of the parent atom. The NH_3_ hydrogen atoms were also found in a difference Fourier map, but were subsequently constrained to ride on their parent atom, with N—H = 0.91 Å and U_iso_ = 1.5U_eq_ (parent atom). The other hydrogen atoms were positioned geometrically and constrained to ride on their parent atoms, with C—H = 0.99 and U_iso_ = 1.2U_eq_(parent atom).

### Crystal data

**Table:**

(NH_4_O)_2_[PtCl_6_](C_12_H_24_O_6_)_2_	*F*(000) = 4048
*M**_r_* = 1004.50	*D*_x_ = 1.733 Mg m^−^^3^
Orthorhombic, *F**d**d*2	Mo *K*α radiation, λ = 0.71073 Å
Hall symbol: F 2 -2d	Cell parameters from 9098 reflections
*a* = 29.6079 (10) Å	θ = 2.6–30.0°
*b* = 30.5302 (10) Å	µ = 4.12 mm^−^^1^
*c* = 8.5175 (3) Å	*T* = 100 K
*V* = 7699.3 (5) Å^3^	Needle, yellow
*Z* = 8	0.48 × 0.12 × 0.11 mm

### Data collection

**Table:**

Bruker Kappa APEXII DUO CCD diffractometer	4628 independent reflections
Radiation source: fine-focus sealed tube	4037 reflections with *I* > 2σ(*I*)
Curved graphite crystal monochromator	*R*_int_ = 0.067
Detector resolution: 16 pixels mm^-1^	θ_max_ = 28.3°, θ_min_ = 1.9°
φ scans and ω scans with κ offset	*h* = −39→39
Absorption correction: multi-scan (*SADABS*; Sheldrick, 2008*a*)	*k* = −40→40
*T*_min_ = 0.241, *T*_max_ = 0.664	*l* = −10→11
29930 measured reflections	

### Refinement

**Table:**

Refinement on *F*^2^	Secondary atom site location: difference Fourier map
Least-squares matrix: full	Hydrogen site location: inferred from neighbouring sites
*R*[*F*^2^ > 2σ(*F*^2^)] = 0.033	H-atom parameters constrained
*wR*(*F*^2^) = 0.054	*w* = 1/[σ^2^(*F*_o_^2^) + 25.8438*P*] where *P* = (*F*_o_^2^ + 2*F*_c_^2^)/3
*S* = 1.13	(Δ/σ)_max_ = 0.001
4628 reflections	Δρ_max_ = 1.49 e Å^−^^3^
215 parameters	Δρ_min_ = −1.23 e Å^−^^3^
1 restraint	Absolute structure: Flack (1983), 2066 Friedel pairs
Primary atom site location: structure-invariant direct methods	Absolute structure parameter: 0.035 (6)

### Special details

**Table:**

Geometry. All e.s.d.'s (except the e.s.d. in the dihedral angle between two l.s. planes) are estimated using the full covariance matrix. The cell e.s.d.'s are taken into account individually in the estimation of e.s.d.'s in distances, angles and torsion angles; correlations between e.s.d.'s in cell parameters are only used when they are defined by crystal symmetry. An approximate (isotropic) treatment of cell e.s.d.'s is used for estimating e.s.d.'s involving l.s. planes.
Refinement. Refinement of *F*^2^ against ALL reflections. The weighted *R*-factor *wR* and goodness of fit *S* are based on *F*^2^, conventional *R*-factors *R* are based on *F*, with *F* set to zero for negative *F*^2^. The threshold expression of *F*^2^ > σ(*F*^2^) is used only for calculating *R*-factors(gt) *etc*. and is not relevant to the choice of reflections for refinement. *R*-factors based on *F*^2^ are statistically about twice as large as those based on *F*, and *R*- factors based on ALL data will be even larger.

### Fractional atomic coordinates and isotropic or equivalent isotropic displacement parameters (Å^2^)

**Table:**

	*x*	*y*	*z*	*U*_iso_*/*U*_eq_	
Pt1	0.2500	0.2500	0.34985 (5)	0.01233 (5)	
Cl1	0.2500	0.2500	0.0765 (3)	0.0260 (7)	
Cl2	0.2500	0.2500	0.6231 (3)	0.0178 (6)	
Cl3	0.22293 (3)	0.32131 (3)	0.35338 (17)	0.0194 (2)	
Cl4	0.17574 (3)	0.22593 (3)	0.34620 (17)	0.0196 (2)	
O1	0.11462 (11)	0.22413 (11)	−0.1142 (4)	0.0191 (7)	
O2	0.09346 (12)	0.30857 (12)	−0.2258 (4)	0.0204 (9)	
O3	0.05499 (11)	0.36586 (11)	−0.0014 (4)	0.0176 (7)	
O4	0.01529 (11)	0.33464 (11)	0.2718 (4)	0.0152 (7)	
O5	0.04401 (9)	0.24907 (13)	0.3768 (5)	0.0154 (10)	
O6	0.06925 (10)	0.18918 (10)	0.1471 (3)	0.0159 (7)	
O7	0.13001 (12)	0.30883 (12)	0.1706 (4)	0.0322 (9)	
H7O	0.1485	0.2909	0.2091	0.048*	
C1	0.1225 (2)	0.23742 (17)	−0.2727 (6)	0.0288 (13)	
H1A	0.0953	0.2314	−0.3372	0.035*	
H1B	0.1481	0.2207	−0.3172	0.035*	
C2	0.13288 (19)	0.28539 (18)	−0.2750 (7)	0.0298 (13)	
H2A	0.1583	0.2918	−0.2032	0.036*	
H2B	0.1416	0.2946	−0.3823	0.036*	
C3	0.10008 (17)	0.35477 (16)	−0.2288 (6)	0.0222 (11)	
H3A	0.1054	0.3646	−0.3380	0.027*	
H3B	0.1269	0.3625	−0.1652	0.027*	
C4	0.05936 (15)	0.37703 (15)	−0.1643 (6)	0.0194 (10)	
H4A	0.0624	0.4092	−0.1760	0.023*	
H4B	0.0321	0.3675	−0.2225	0.023*	
C5	0.02007 (16)	0.39000 (15)	0.0749 (5)	0.0196 (11)	
H5A	−0.0098	0.3813	0.0328	0.024*	
H5B	0.0242	0.4218	0.0564	0.024*	
C6	0.02242 (17)	0.38033 (15)	0.2473 (6)	0.0193 (10)	
H6A	0.0524	0.3889	0.2888	0.023*	
H6B	−0.0009	0.3974	0.3037	0.023*	
C7	0.02376 (18)	0.32346 (16)	0.4320 (6)	0.0200 (11)	
H7A	0.0053	0.3422	0.5019	0.024*	
H7B	0.0560	0.3284	0.4572	0.024*	
C8	0.01196 (16)	0.27634 (16)	0.4571 (6)	0.0204 (11)	
H8A	0.0123	0.2696	0.5708	0.025*	
H8B	−0.0188	0.2705	0.4167	0.025*	
C9	0.03121 (15)	0.20407 (14)	0.3860 (5)	0.0166 (10)	
H9A	0.0018	0.1996	0.3330	0.020*	
H9B	0.0280	0.1952	0.4973	0.020*	
C10	0.06677 (15)	0.17702 (15)	0.3083 (5)	0.0159 (10)	
H10A	0.0963	0.1820	0.3598	0.019*	
H10B	0.0592	0.1455	0.3175	0.019*	
C11	0.10475 (15)	0.16589 (16)	0.0690 (5)	0.0191 (11)	
H11A	0.1000	0.1339	0.0802	0.023*	
H11B	0.1342	0.1734	0.1168	0.023*	
C12	0.10464 (18)	0.17826 (17)	−0.1014 (6)	0.0194 (12)	
H12A	0.1276	0.1610	−0.1587	0.023*	
H12B	0.0747	0.1720	−0.1480	0.023*	
N1	0.09142 (12)	0.28600 (12)	0.1164 (5)	0.0180 (8)	
H1C	0.0998	0.2660	0.0429	0.027*	
H1D	0.0716	0.3053	0.0732	0.027*	
H1E	0.0780	0.2720	0.1985	0.027*	

### Atomic displacement parameters (Å^2^)

**Table:**

	*U*^11^	*U*^22^	*U*^33^	*U*^12^	*U*^13^	*U*^23^
Pt1	0.01615 (9)	0.00846 (9)	0.01239 (9)	−0.00288 (16)	0.000	0.000
Cl1	0.0447 (18)	0.0187 (15)	0.0145 (16)	−0.0078 (8)	0.000	0.000
Cl2	0.0220 (14)	0.0191 (14)	0.0123 (14)	−0.0011 (7)	0.000	0.000
Cl3	0.0241 (5)	0.0112 (5)	0.0229 (5)	−0.0006 (4)	−0.0023 (6)	0.0014 (6)
Cl4	0.0179 (5)	0.0172 (5)	0.0237 (5)	−0.0064 (4)	−0.0031 (6)	0.0034 (6)
O1	0.0255 (18)	0.0156 (17)	0.016 (2)	0.0005 (14)	0.0061 (14)	−0.0010 (14)
O2	0.027 (2)	0.016 (2)	0.0184 (18)	−0.0011 (16)	0.0046 (15)	−0.0011 (15)
O3	0.0233 (18)	0.0147 (18)	0.0146 (16)	0.0019 (14)	0.0004 (14)	0.0031 (14)
O4	0.0220 (18)	0.0102 (17)	0.0136 (16)	0.0005 (13)	−0.0005 (13)	−0.0006 (13)
O5	0.0187 (13)	0.0102 (13)	0.017 (3)	0.0021 (17)	0.0054 (14)	−0.0013 (16)
O6	0.0195 (17)	0.0151 (17)	0.0130 (17)	0.0023 (13)	0.0018 (12)	−0.0003 (12)
O7	0.026 (2)	0.022 (2)	0.049 (2)	−0.0009 (16)	−0.0164 (17)	0.0057 (18)
C1	0.039 (3)	0.027 (3)	0.020 (3)	0.004 (2)	0.013 (2)	0.000 (2)
C2	0.033 (3)	0.030 (3)	0.026 (3)	0.005 (3)	0.012 (3)	0.004 (3)
C3	0.032 (3)	0.017 (3)	0.017 (2)	−0.008 (2)	0.000 (2)	0.003 (2)
C4	0.029 (2)	0.014 (2)	0.015 (2)	−0.0047 (18)	−0.005 (2)	0.004 (2)
C5	0.024 (2)	0.010 (2)	0.024 (3)	0.0044 (18)	−0.001 (2)	0.003 (2)
C6	0.026 (3)	0.011 (2)	0.021 (3)	0.000 (2)	0.003 (2)	0.000 (2)
C7	0.027 (3)	0.017 (3)	0.016 (3)	0.000 (2)	0.003 (2)	−0.002 (2)
C8	0.025 (3)	0.018 (3)	0.018 (2)	0.0057 (19)	0.007 (2)	0.002 (2)
C9	0.020 (2)	0.011 (2)	0.018 (3)	−0.0035 (18)	0.0008 (18)	0.0010 (18)
C10	0.019 (2)	0.010 (2)	0.018 (3)	−0.0010 (18)	−0.0015 (18)	0.0013 (18)
C11	0.021 (2)	0.016 (2)	0.021 (3)	0.0044 (18)	0.001 (2)	−0.0034 (19)
C12	0.023 (3)	0.015 (3)	0.019 (3)	0.000 (2)	0.001 (2)	−0.006 (2)
N1	0.0226 (19)	0.0145 (19)	0.017 (2)	−0.0013 (15)	0.0002 (19)	0.0010 (19)

### Geometric parameters (Å, º)

**Table:**

Pt1—Cl4	2.3184 (10)	C3—H3B	0.9900
Pt1—Cl4^i^	2.3184 (10)	C4—H4A	0.9900
Pt1—Cl3^i^	2.3202 (10)	C4—H4B	0.9900
Pt1—Cl3	2.3202 (10)	C5—C6	1.499 (7)
Pt1—Cl2	2.327 (3)	C5—H5A	0.9900
Pt1—Cl1	2.328 (3)	C5—H5B	0.9900
O1—C1	1.428 (6)	C6—H6A	0.9900
O1—C12	1.435 (6)	C6—H6B	0.9900
O2—C3	1.424 (6)	C7—C8	1.496 (7)
O2—C2	1.428 (6)	C7—H7A	0.9900
O3—C5	1.427 (5)	C7—H7B	0.9900
O3—C4	1.435 (6)	C8—H8A	0.9900
O4—C6	1.426 (5)	C8—H8B	0.9900
O4—C7	1.429 (5)	C9—C10	1.493 (6)
O5—C9	1.427 (6)	C9—H9A	0.9900
O5—C8	1.436 (6)	C9—H9B	0.9900
O6—C10	1.425 (5)	C10—H10A	0.9900
O6—C11	1.432 (5)	C10—H10B	0.9900
O7—N1	1.416 (5)	C11—C12	1.500 (6)
O7—H7O	0.8400	C11—H11A	0.9900
C1—C2	1.497 (7)	C11—H11B	0.9900
C1—H1A	0.9900	C12—H12A	0.9900
C1—H1B	0.9900	C12—H12B	0.9900
C2—H2A	0.9900	N1—H1C	0.9100
C2—H2B	0.9900	N1—H1D	0.9100
C3—C4	1.489 (6)	N1—H1E	0.9100
C3—H3A	0.9900		
			
Cl4—Pt1—Cl4^i^	178.46 (7)	C6—C5—H5B	110.1
Cl4—Pt1—Cl3^i^	91.73 (4)	H5A—C5—H5B	108.4
Cl4^i^—Pt1—Cl3^i^	88.29 (4)	O4—C6—C5	109.2 (4)
Cl4—Pt1—Cl3	88.29 (4)	O4—C6—H6A	109.8
Cl4^i^—Pt1—Cl3	91.73 (4)	C5—C6—H6A	109.8
Cl3^i^—Pt1—Cl3	178.52 (7)	O4—C6—H6B	109.8
Cl4—Pt1—Cl2	90.77 (4)	C5—C6—H6B	109.8
Cl4^i^—Pt1—Cl2	90.77 (4)	H6A—C6—H6B	108.3
Cl3^i^—Pt1—Cl2	89.26 (4)	O4—C7—C8	109.0 (4)
Cl3—Pt1—Cl2	89.26 (4)	O4—C7—H7A	109.9
Cl4—Pt1—Cl1	89.23 (4)	C8—C7—H7A	109.9
Cl4^i^—Pt1—Cl1	89.23 (4)	O4—C7—H7B	109.9
Cl3^i^—Pt1—Cl1	90.74 (4)	C8—C7—H7B	109.9
Cl3—Pt1—Cl1	90.74 (4)	H7A—C7—H7B	108.3
Cl2—Pt1—Cl1	180.000 (1)	O5—C8—C7	109.6 (4)
C1—O1—C12	112.5 (4)	O5—C8—H8A	109.8
C3—O2—C2	111.9 (4)	C7—C8—H8A	109.8
C5—O3—C4	112.5 (3)	O5—C8—H8B	109.8
C6—O4—C7	110.3 (4)	C7—C8—H8B	109.8
C9—O5—C8	110.9 (3)	H8A—C8—H8B	108.2
C10—O6—C11	110.8 (3)	O5—C9—C10	108.7 (4)
N1—O7—H7O	109.5	O5—C9—H9A	109.9
O1—C1—C2	108.9 (4)	C10—C9—H9A	109.9
O1—C1—H1A	109.9	O5—C9—H9B	109.9
C2—C1—H1A	109.9	C10—C9—H9B	109.9
O1—C1—H1B	109.9	H9A—C9—H9B	108.3
C2—C1—H1B	109.9	O6—C10—C9	108.6 (4)
H1A—C1—H1B	108.3	O6—C10—H10A	110.0
O2—C2—C1	108.2 (4)	C9—C10—H10A	110.0
O2—C2—H2A	110.1	O6—C10—H10B	110.0
C1—C2—H2A	110.1	C9—C10—H10B	110.0
O2—C2—H2B	110.1	H10A—C10—H10B	108.3
C1—C2—H2B	110.1	O6—C11—C12	108.8 (4)
H2A—C2—H2B	108.4	O6—C11—H11A	109.9
O2—C3—C4	109.5 (4)	C12—C11—H11A	109.9
O2—C3—H3A	109.8	O6—C11—H11B	109.9
C4—C3—H3A	109.8	C12—C11—H11B	109.9
O2—C3—H3B	109.8	H11A—C11—H11B	108.3
C4—C3—H3B	109.8	O1—C12—C11	108.6 (4)
H3A—C3—H3B	108.2	O1—C12—H12A	110.0
O3—C4—C3	108.7 (4)	C11—C12—H12A	110.0
O3—C4—H4A	109.9	O1—C12—H12B	110.0
C3—C4—H4A	109.9	C11—C12—H12B	110.0
O3—C4—H4B	109.9	H12A—C12—H12B	108.4
C3—C4—H4B	109.9	O7—N1—H1C	109.5
H4A—C4—H4B	108.3	O7—N1—H1D	109.5
O3—C5—C6	108.1 (4)	H1C—N1—H1D	109.5
O3—C5—H5A	110.1	O7—N1—H1E	109.5
C6—C5—H5A	110.1	H1C—N1—H1E	109.5
O3—C5—H5B	110.1	H1D—N1—H1E	109.5

Symmetry code: (i) −*x*+1/2, −*y*+1/2, *z*.

### Hydrogen-bond geometry (Å, º)

**Table:**

*D*—H···*A*	*D*—H	H···*A*	*D*···*A*	*D*—H···*A*
O7—H7*O*···Cl4	0.84	2.44	3.237 (4)	159
O7—H7*O*···Cl3	0.84	2.69	3.184 (4)	119
N1—H1*C*···O1	0.91	1.90	2.811 (5)	177
N1—H1*D*···O3	0.91	2.02	2.849 (5)	152
N1—H1*D*···O4	0.91	2.54	3.006 (5)	113
N1—H1*E*···O5	0.91	1.95	2.856 (6)	172
N1—H1*E*···O6	0.91	2.58	3.039 (5)	112
